# Serum Biomarker Profile Including CCL1, CXCL10, VEGF, and Adenosine Deaminase Activity Distinguishes Active From Remotely Acquired Latent Tuberculosis

**DOI:** 10.3389/fimmu.2021.725447

**Published:** 2021-10-07

**Authors:** Eveline M. Delemarre, Laura van Hoorn, Aik W. J. Bossink, Julia Drylewicz, Simone A. Joosten, Tom H. M. Ottenhoff, Onno W. Akkerman, Delia Goletti, Elisa Petruccioli, Assunta Navarra, Brigitte T. A. van den Broek, Sanne P. A. Paardekooper, Ineke van Haeften, Leo Koenderman, Jan-Willem J. Lammers, Steven F. T. Thijsen, Regina W. Hofland, Stefan Nierkens

**Affiliations:** ^1^ Center for Translational Immunology (CTI), University Medical Center Utrecht, Utrecht, Netherlands; ^2^ Platform Immune Monitoring (PIM), University Medical Center Utrecht, Utrecht, Netherlands; ^3^ Department of Respiratory Medicine and Tuberculosis, University Medical Center Utrecht, Utrecht, Netherlands; ^4^ Department of Respiratory Medicine and Tuberculosis, Diakonessenhuis, Utrecht, Netherlands; ^5^ Department of Infectious Diseases, Leiden University Medical Center, Leiden, Netherlands; ^6^ Department of Respiratory Medicine and Tuberculosis, University Medical Center Groningen, Groningen, Netherlands; ^7^ Translational Research Unit, Department of Epidemiology and Preclinical Research, National Institute for Infectious Diseases-IRCCS L. Spallanzani, Rome, Italy; ^8^ Department of Tuberculosis, Municipal Public Health Service, Utrecht, Netherlands; ^9^ Department of Medical Microbiology and Immunology, Diakonessenhuis, Utrecht, Netherlands

**Keywords:** active tuberculosis (ATB), biomarker, diagnosis, adenosine deaminase activity (ADA), CCL1, vascular endothelial growth factor (VEGF), CXCL10 (IP-10), latent tuberculosis infection (LTBI)

## Abstract

**Introduction:**

There is an urgent medical need to differentiate active tuberculosis (ATB) from latent tuberculosis infection (LTBI) and prevent undertreatment and overtreatment. The aim of this study was to identify biomarker profiles that may support the differentiation between ATB and LTBI and to validate these signatures.

**Materials and Methods:**

The discovery cohort included adult individuals classified in four groups: ATB (n = 20), LTBI without prophylaxis (untreated LTBI; n = 20), LTBI after completion of prophylaxis (treated LTBI; n = 20), and healthy controls (HC; n = 20). Their sera were analyzed for 40 cytokines/chemokines and activity of adenosine deaminase (ADA) isozymes. A prediction model was designed to differentiate ATB from untreated LTBI using sparse partial least squares (sPLS) and logistic regression analyses. Serum samples of two independent cohorts (national and international) were used for validation.

**Results:**

sPLS regression analyses identified C-C motif chemokine ligand 1 (CCL1), C-reactive protein (CRP), C-X-C motif chemokine ligand 10 (CXCL10), and vascular endothelial growth factor (VEGF) as the most discriminating biomarkers. These markers and ADA(2) activity were significantly increased in ATB compared to untreated LTBI (p ≤ 0.007). Combining CCL1, CXCL10, VEGF, and ADA2 activity yielded a sensitivity and specificity of 95% and 90%, respectively, in differentiating ATB from untreated LTBI. These findings were confirmed in the validation cohort including remotely acquired untreated LTBI participants.

**Conclusion:**

The biomarker signature of CCL1, CXCL10, VEGF, and ADA2 activity provides a promising tool for differentiating patients with ATB from non-treated LTBI individuals.

## Introduction

Tuberculosis (TB) is an infectious disease caused by a species of the *Mycobacterium tuberculosis* (*Mtb*) complex, most commonly the bacillus *Mtb (* 1). TB represents a major global health problem with an estimated incidence of 10 million and a mortality of 1.4 million persons in 2019 (2). Patients with TB can infect 10–15 close contacts within 1 year, and approximately 45% of infected patients will die without proper treatment (2). It is estimated that one-fourth of the world’s population suffers from a latent TB infection (LTBI) (3–5) that (in the absence of a gold standard) is defined by the World Health Organization as “a state of persistent immune response to *Mtb* without clinical evidence of active TB disease” (2). This definition includes a continuum of stages ranging from clearance of the pathogen to dormant, but live tuberculous mycobacteria (6, 7). The lack of evidence for the presence of live tuberculous mycobacteria in LTBI generates clinical unanswered questions in general practice and research. The lifetime risk of reactivation and progression from LTBI to active TB (ATB) is 5%–10% and mostly occurs in the first years following infection (8). In low-burden countries, reactivation accounts for approximately 80% of new ATB cases, which could be averted by timely diagnosis and preventive treatment with an efficacy of 60%–90% (8). The tuberculin skin test (TST) and interferon gamma release assay (IGRA) are currently used for LTBI diagnosis, although they do not allow discriminating ATB from LTBI and predict with low accuracy the risk of progression to ATB (2, 9–13).

The clinical presentation of ATB is diverse; definite diagnosis can be challenging because of limited sensitivity of the nucleic acid amplification test and *Mtb* cultures, especially in extrapulmonary forms of ATB. The GeneXpert^®^ MTB/RIF sputum test has a higher sensitivity compared to that of *Mtb* cultures (14, 15). The newer Xpert Ultra^®^ test increased the sensitivity for paucibacillary disease. However, both tests are sputum based, which causes problems for patients who are not able to produce sufficient sputum production, which is more often seen in children and patients with extrapulmonary disease (16). As a result, discrimination between ATB and LTBI can be difficult in clinical practice, leading to undertreatment of patients with ATB and overtreatment of patients with LTBI (4, 6). Despite optimal diagnostic facilities, microbiological confirmation is lacking in about 30%–35% of the patients with ATB in the Netherlands (17). Therefore, the aim of this study was to identify novel biomarker profiles in serum to differentiate between ATB and LTBI.

Infection with *Mtb* causes a cascade of immune responses, including production of cytokines/chemokines and generation of enzyme activities important to limit bacterial growth, regulate inflammation, and seal off the infection by creating a granuloma (18). These mechanisms may result in systemic fingerprints indicating active *Mtb* replication and therefore potentially useful in distinguishing ATB from LTBI. Activity measurement of the enzyme adenosine deaminase (ADA) and its two isoforms (ADA1 and ADA2) in a variety of biological fluids has been suggested to aid in the diagnosis of ATB; however, ADA activity in serum has never been used to distinguish ATB from LTBI (19–23). Several studies exploring the use of inflammatory serum cytokines involved in *Mtb* infection have been performed (24–29), with inconsistent conclusions for cytokine production in *Mtb-*specific stimulated CD4^+^ and CD8^+^ T cells (30–45). Most of these studies showed that the discriminative value of a single marker is limited. Here, we report that a specific combination of serum markers has promising diagnostic potential to distinguish ATB from untreated LTBI.

## Materials and Methods

### Study Participants

In the discovery cohort, 80 HIV-negative adults were enrolled in this cross-sectional study from December 2015 until January 2017 through the Public Health Service of Utrecht and the Diakonessenhuis (Utrecht, Netherlands). Individuals were classified into four groups: ATB (n = 20) with pulmonary and/or extrapulmonary disease, latent TB without prophylactic treatment (untreated LTBI; n = 20), latent TB individuals after completion of prophylactic treatment (treated LTBI; n = 20), and healthy controls (HCs; n = 20). ATB patients were microbiologically diagnosed according to the American Thoracic Society guidelines and were included before or within 8 days after start of treatment (46). Participants were included in the LTBI group in case of a positive TST and/or IGRA in the absence of clinical evidence of TB and a likely recent infection in the last 2 years, as determined by documented contact with an infectious case and absence of documented TB contact previously. Prophylactic treatment entailed 3 months of rifampicin and isoniazid or 6 months of isoniazid. LTBI individuals who completed prophylactic therapy were indicated as “LTBI-cured,” here referred to as treated LTBI. HCs were included as a reference group and consisted of healthcare workers with no history of or contact with TB who were screened annually for TB and they all had a recent negative IGRA. All enrolled participants signed a written informed consent and completed a questionnaire regarding their medical history, history of TB, smoking status, and demographics (**Table 1**). This study was approved by the Medical Research Ethics Committees-United (NL53628.100.15) and the Board of Directors of the Public Health and Diakonessenhuis, Utrecht, Netherlands.

**Table 1:** Baseline characteristics of the discovery cohort.

	ATB (n = 20)	Untreated -LTBI (n = 20)	Treated LTBI (n = 20)	HC (n = 20)
**Male sex, n (%)**	11 (55)	8 (40)	7 (35)	8 (40)
**Age (years), mean ± SD**	48 ± 13	36 ± 13	36 ± 13	37 ± 11
**Pulmonary TB, n (%)**	11 (55)	0	0	0
TB high burden countries^*^, n (%)	10 (50)	4 (20)	0 (0)	0 (0)
**TB in history, n (%)**	1 (5)	0 (0)	0 (0)	0 (0)
**Prior BCG vaccination, n (%)**	7 (35)	7 (35)	2 (10)	3 (15)
**Missing, n (%)**	5 (25)	3 (15)	1 (5)	0 (0)
**Smoking or in history, n (%)**	11 (55)	8 (40)	10 (50)	2 (10)
**Diabetes, n (%)**	3 (15)	2 (10)	0 (0)	0 (0)
**Kidney disease, n (%)**	1 (5)	1 (5)	0 (0)	0 (0)
**Immunosuppressive medication, n (%)**	2 (10)	0 (0)	0 (0)	0 (0)

ATB, active tuberculosis; Untreated LTBI, latent tuberculosis infection without prophylaxis; Treated LTBI, latent tuberculosis infection after completion of prophylaxis; HC, healthy control; n, sample size; SD, standard deviation; TB, tuberculosis; BCG, Bacillus Calmette-Guérin.

*Defined as a country of origin with a TB incidence of more than 50 cases per 100,000 inhabitants annually.

For the validation, two independent cohorts were included. One cohort, collected by the Leiden University Medical Center (LUMC, Leiden, Netherlands), consisted of 12 ATB patients (treated in a specialized TB clinic, Groningen, Netherlands) and 20 untreated LTBI individuals who all had a positive IGRA. The untreated LTBI individuals within this cohort have been previously described (47). A second cohort (National Institute of Infectious Diseases, Rome, Italy) included 31 ATB patients and 20 untreated LTBI individuals with positive IGRA who were sharing their households with ATB patients no more than 3 months before inclusion in the study. These Italian untreated LTBI individuals are different compared to the Dutch untreated LTBI individuals because the Dutch individuals were the result of a contact investigation, employment verification, or periodic screening and probably had a recent infection in the last 2 years. Therefore, we will refer to the Italian untreated LTBI individuals who all shared their households with ATB patients before inclusion in the study as HH-LTBI. This cohort has been previously described (35). All participants signed a written informed consent before participation. For the Leiden cohort, the study was approved by the Medical Ethics Committee of LUMC (METC project number P07.048). The Italian study was approved by the Ethical Committee of the L. Spallanzani National Institute of Infectious diseases (INMI approval numbers 02/2007 and 72/2015). Inclusion strategies for both ATB and untreated LTBI groups of the validation cohorts were similar as those for the discovery cohort.

### Measurement of Serum Mediators

Levels of 40 cytokines/chemokines were measured in serum samples of the discovery cohort using an in-house developed and validated (ISO9001) multiplex immunoassay based on xMAP technology (Luminex Corporation, Austin, USA) (**Supplementary Table S1**). The assay was performed as previously described (48, 49). In short, a specific heterophilic immunoglobulins were preabsorbed from all samples with heteroblock (Omega Biologicals, Bozeman, MT, USA). Next, samples were incubated with antibody-conjugated MagPlex microspheres for 1 h at room temperature with continuous shaking, followed by 1-h incubation with biotinylated antibodies and 10-min incubation with phycoerythrin-conjugated streptavidin diluted in high-performance ELISA buffer (HPE, Sanquin, Netherlands). Acquisition was performed with the Bio-Rad FlexMAP3D (Bio-Rad Laboratories, Hercules, CA, USA) in combination with xPONENT software version 4.2 (Luminex). Data were analyzed by five-parametric curve fitting using Bio-Plex Manager software, version 6.1.1 (Bio-Rad). Serum samples were diluted to measure S100A8, SAA1, Angiotensin Converting Enzyme (ACE), and C-reactive protein (CRP) in detectable range: 1:10, 1:100, 1:100, 1:1,000, respectively. Imputation of the lower limit of quantification (LLOQ) divided by 2 was performed for samples with concentrations under the detection limit. Samples with concentrations above the detection limit, the upper limit of quantification (ULOQ) multiplied by 2 was imputed, except for the diluted cytokines. For the diluted cytokines, the highest result of the extrapolated values was used to impute for results above the detection limit.

Total ADA and ADA2 activities were determined using an ADA assay kit (Diazyme Laboratories, Poway, CA, USA) according to manufacturer’s instructions with minor modifications. In short, to measure ADA2 activity, 0.1 μM erythro-9 (2-hydroxy-3-nonyl) adenine (EHNA) was added to inhibit ADA1 activity (50). Activity measurements were performed using the SpectraMax M2e Plate Reader (Molecular Devices, Sunnyvale, CA, USA). The activity of ADA1 was calculated by subtracting the ADA2 activity from the total ADA activity. ADA activity was expressed in U/L.

### Statistical Analysis

Baseline characteristics were reported as mean and standard deviation (SD) for continuous variables and as absolute numbers and percentages for binary variables. Comparisons between patient groups in the discovery cohort were performed using the Kruskal–Wallis test for continuous variables and Fisher–Freeman–Halton exact test for binary variables. Within the validation cohorts, comparisons were made between the ATB and LTBI groups using the Mann–Whitney U test for continuous variables and Fisher’s exact test for binary variables. Sparse partial least squares regression (sPLS) analysis was applied on the dataset including the 40 measured serum mediators obtained for the discovery cohort to identify serum markers that could be used to differentiate ATB from untreated LTBI (R package *MixOmics*) (51). The hereby selected markers, total ADA, ADA1, and ADA2 activities were compared between the four patient groups of the discovery cohort (ATB, untreated LTBI, treated LTBI, and HC) and the two groups in the validation cohorts (ATB vs. untreated LTBI/HH-LTBI) using Mann–Whitney tests. Logistic regression analyses including backward stepwise selection were used to acquire a model able to classify individuals into ATB from untreated LTBI in the discovery cohort (R package *stats*) (52). The model (i.e., formula based on the selected biomarkers) with the lowest Akaike information criterion (AIC) was kept for further analyses. All variables measured by Luminex were logarithmically transformed (log10). Receiver operating characteristic (ROC) analyses and area under the curve (AUC) were obtained for all markers separately and the final models. The two biomarker signatures were applied on the datasets obtained for the two independent validation cohorts using the logistic regression coefficients and optimal cutoff found for the discovery cohort (R package *InformationValue*) (53). The optimal cutoff is obtained when the most participants are correctly identified, thereby achieving the highest sensitivity and specificity. For all three patient cohorts, sensitivity and specificity in discriminating ATB from untreated LTBI were calculated using the biomarker combinations. Results for the 40 measured cytokines/chemokines per subgroup of the discovery cohort were reported as median and interquartile range (IQR), and raw data are available (**Supplementary Data 1**). SPSS software (version 22.0) and R Project Software (version 3.2.0) were used for statistical analysis. p-values <0.05 were considered statistically significant.

## Results

### Study Participants

Within the discovery cohort, participants in the ATB group were significantly older compared to the untreated LTBI, treated LTBI, and HC groups (p < 0.005; **Table 1**) and more often originating from a TB-endemic country (defined as a country of origin with a TB incidence of more than 50 cases per 100,000 inhabitants annually) compared to individuals in the treated LTBI and HC groups (p = 0.0001). Smoking was more frequent in the ATB and treated LTBI groups compared to HCs (p = 0.006 and p = 0.014, respectively). Other baseline characteristics showed no significant differences between the four groups (**Table 1**). In the Leiden and Italy cohorts, age did not significantly differ between the ATB and untreated LTBI/HH-LTBI individuals (**Table 2**). Within the Italian cohort, more males were included in the ATB group compared to the HH-LTBI group (p = 0.046) and more ATB patients originated from a TB-endemic country (p = 0.004).

**Table 2:** Baseline characteristics of the validation cohorts.

	ATB	Untreated LTBI
**Leiden (national cohort)**	n = 12	n = 20
Male sex, n (%)	9 (75%)	11 (55%)
Age (years), mean ± SD	37 ± 23	35 ± 11
Pulmonary TB, n (%)	10 (83%)	0
TB high burden countries*, n (%)	5 (42%)	7 (35%)
	**ATB**	**HH-LTBI**
**Italy (international cohort)**	n = 31	n = 20
Male sex, n (%)	17 (55%)	5 (25%)
Age (years), mean ± SD	40 ± 14	44 ± 16
Pulmonary TB, n (%)	31 (100%)	0
TB high burden countries*, n (%)	21 (68%)	2 (11%)

ATB, active tuberculosis; Untreated LTBI, latent tuberculosis infection without prophylaxis; Households, individuals diagnosed with untreated LTBI who shared their households with ATB patient before inclusion within the cohort; n, sample size; SD, standard deviation; TB, tuberculosis.

*Defined as a country of origin with a TB incidence of more than 50 cases per 100.000 citizens annually.

### Identification of Serum Biomarkers for Distinguishing ATB From Untreated LTBI

**Figure 1:**
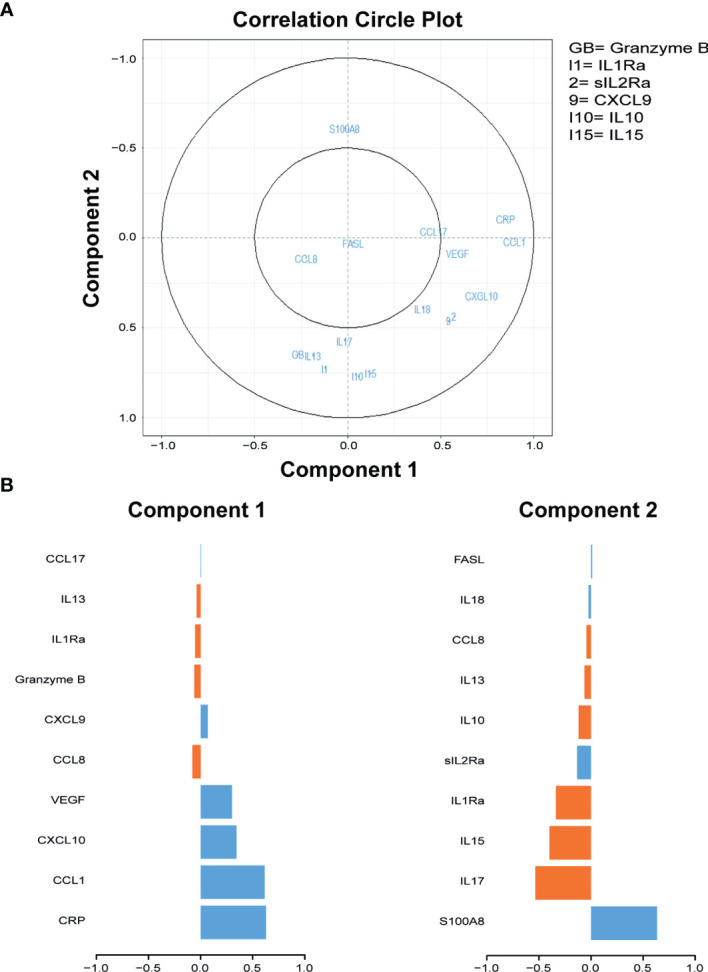
Sparse partial least squares regression analyses of 40 cytokines measured in serum samples of individuals diagnosed with ATB and untreated LTBI. **(A)** Correlation circle plots representing the correlations between the cytokines and the first two components of the sPLS. A correlation circle is a two-dimensional plot helping the interpretation of the correlations between the initial variables (i.e., the cytokines) and the new variables (i.e., the components) that are combinations of the first ones: the closer a variable is to an axis and to the circle, the higher the correlation with the corresponding component will be. It is also a way to plot the correlations between the cytokines: if two cytokines are in opposite quarters, they are negatively correlated; if two cytokines are perpendicular, then their correlation is close to 0; if two variables are close, their correlation is close to 1. Top 10 cytokines of the first two components are shown in the graph. GB, Granzyme B; I1, Il1Ra; 2, sIL2Ra; 9, CXCL9; I10, IL-10; I15, IL-15. **(B)** The contribution (i.e., the correlation) of the top 10 cytokines for components 1 and 2. The blue bars represent ATB, and the orange bars represent untreated LTBI. ATB, active tuberculosis; untreated LTBI, latent tuberculosis infection without prophylaxis; sPLS, sparse partial least squares.

**Figure 2:**
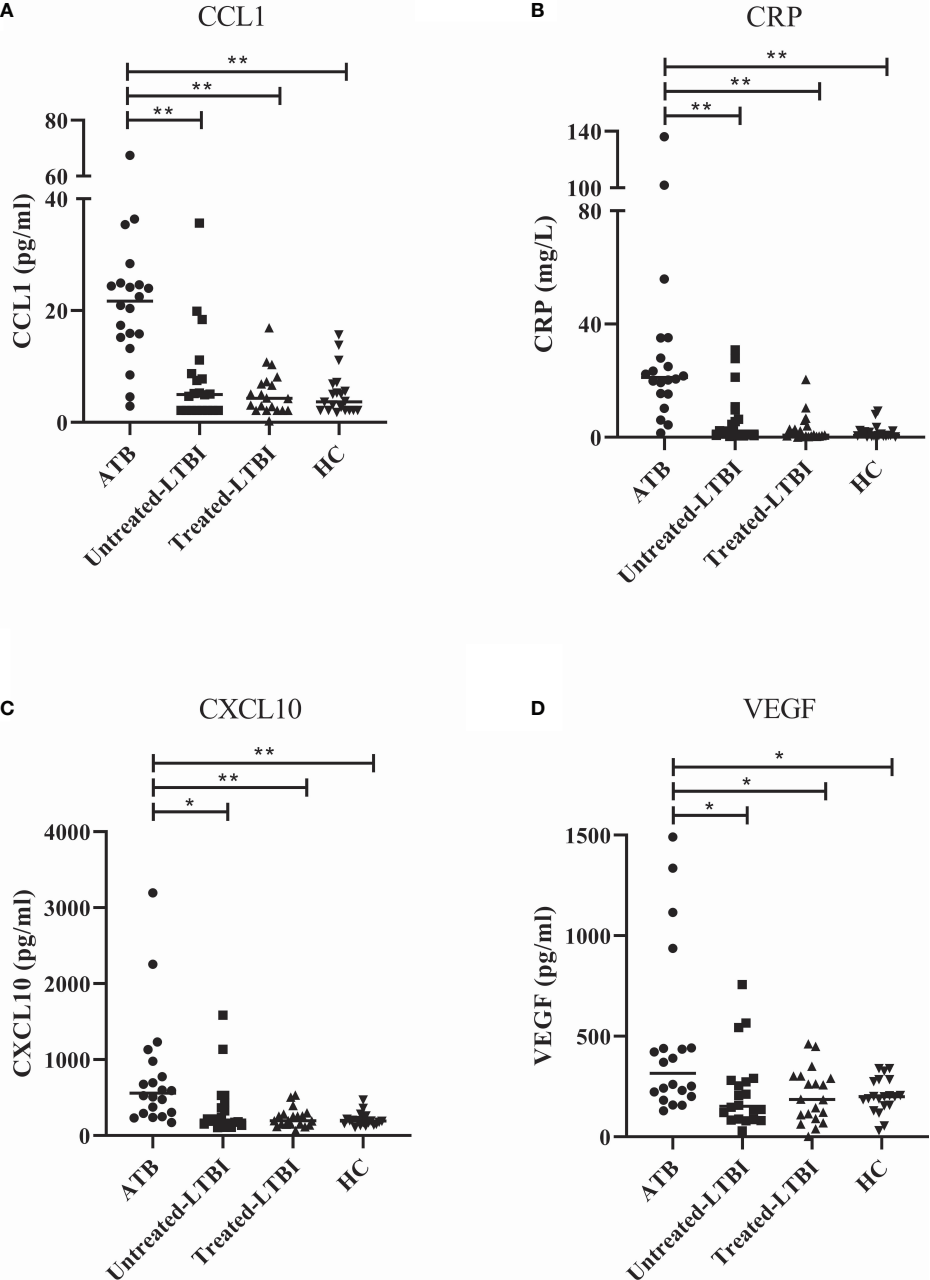
Serum levels of CCL1 **(A)**, CRP **(B)**, CXCL10 **(C)**, and VEGF **(D)** in individuals with ATB, untreated LTBI, and treated LTBI and HCs. All groups were compared for each marker using Mann–Whitney U tests. In panels **(A–D)**, the filled dots represent participants with ATB, the filled squares represent the participants with untreated LTBI, the filled triangles represent the participants with treated LTBI, and the filled inversed triangles represent the HCs. The horizontal line in each study group represents the median concentration. ATB, active tuberculosis; CCL1, C-C motif chemokine ligand 1; CRP, C-reactive protein; CXCL10, C-X-C motif chemokine ligand 10; HC, healthy controls; untreated LTBI, latent tuberculosis infection without prophylaxis; treated LTBI, latent tuberculosis infection after completion of prophylaxis; VEGF, vascular endothelial growth factor. * 0.001 < p < 0.05, ** p < 0.001.

To identify biomarkers that may aid in differentiating ATB from untreated LTBI, 40 cytokines/chemokines and ADA activity including isoforms were measured in serum samples of participants included in the discovery cohort. sPLS analyses and Mann–Whitney tests demonstrated that C-C motif chemokine ligand 1 (CCL1), CRP, C-X-C motif chemokine ligand 10 (CXCL10), and vascular endothelial growth factor (VEGF) were the strongest differentiating markers between ATB and untreated LTBI individuals (**Figures 1**, **2**; p = 0.0001, p = 0.007, p = 0.0001, and p = 0.0001, respectively). Moreover, these markers were significantly increased in the sera of ATB patients compared to treated LTBI individuals and HCs (**Figure 2**). Within the untreated LTBI, for each marker, three amples seem to have higher levels of each protein compared to the other samples. These samples do not belong to the same participants. Comparing cytokine expression between pulmonary and extrapulmonary TB in a subgroup analysis showed no differences (**Supplementary Figure S1**). Results for all measured cytokines/chemokines are shown in **Supplementary Table S1**.

**Figure 3:**
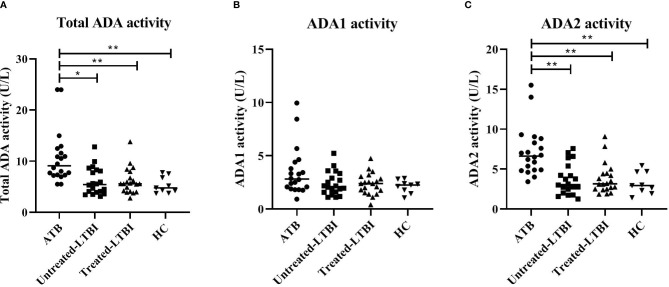
ADA activity levels in serum of individuals with ATB, untreated LTBI, and treated LTBI and HCs. Serum levels for total ADA **(A)**, ADA1 **(B)**, and ADA2 **(C)** activity levels are shown. All groups were compared for each marker using Mann–Whitney U tests. In panels **(A–C)**, the filled dots represent participants with ATB, the filled squares represent the participants with untreated LTBI, the filled triangles represent the participants with treated-LTBI, and the filled inversed triangles represent the HCs. The horizontal line in each study group represents the median concentration. ADA, adenosine deaminase; ADA1, adenosine deaminase-1; ADA2, adenosine deaminase-2; ATB, active tuberculosis; LTBI, latent tuberculosis infection. * 0.001 < p < 0.05, ** p < 0.001.

Furthermore, ADA activity was significantly increased in the ATB group compared to untreated LTBI, treated LTBI, and HC groups (p ≤ 0.001; **Figure 3**). The isoform ADA2 was responsible for this increase (ADA1 showed no significant between-group differences).

**Figure 4:**
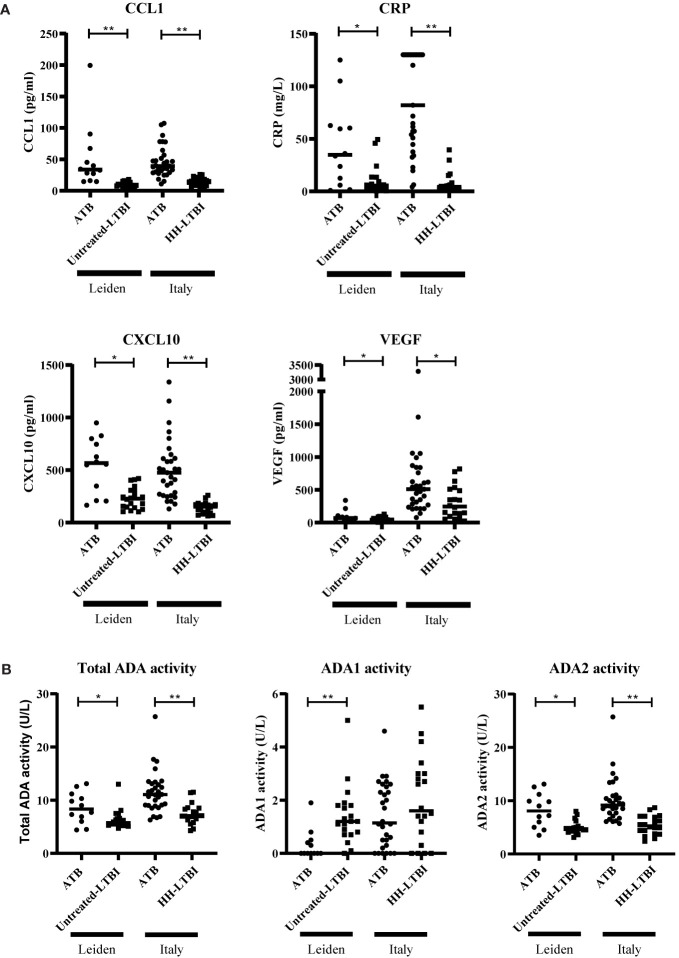
Serum levels of CCL1, CRP, CXCL10, VEGF, and ADA activity differently expressed in patients with ATB compared to untreated LTBI individuals in two independent cohorts. Serum levels of CCL1, CRP, CXCL10, VEGF **(A)**, and ADA activity **(B)** are shown. The two patient groups per cohort were compared for each marker using Mann–Whitney U tests. Filled dots represent patients with ATB, and filled squares represent individuals with untreated LTBI or households of ATB patients diagnosed with untreated LTBI/untreated HH-LTBI. Median concentration is depicted with horizontal line in each group. ADA, adenosine deaminase; ATB, active tuberculosis; CCL1, C-C motif chemokine ligand 1; CRP, C-reactive protein; CXCL10, C-X-C motif chemokine ligand 10; LTBI, latent tuberculosis infection; VEGF, vascular endothelial growth factor. * 0.001 < p < 0.05, ** p < 0.001.

To investigate whether CCL1, CXCL10, CRP, VEGF, and ADA(2) activity are potential biomarkers, these markers were measured in two independent validation cohorts. For one patient within the “Italian” ATB group, ADA activity measurement was not performed. In both cohorts, CCL1, CXCL10, CRP, and VEGF levels were significantly increased in the sera of ATB patients compared to untreated LTBI or HH-LTBI individuals (**Figure 4A**). In addition, total ADA activity was also significantly increased in the ATB patients due to increased activity of ADA2 (**Figure 4B**). Both validation cohorts confirmed the different expression patterns of CCL1, CRP, CXCL10, VEGF, and ADA activity in sera of ATB patients compared to untreated LTBI/HH-LTBI individuals. Hence, we identified five potential biomarkers that may help discriminate between ATB and untreated LTBI individuals.

### Prediction Model to Discriminate ATB From Untreated LTBI

Subsequently, we explored whether these biomarkers could be used for differentiating between ATB and untreated LTBI individuals. Logistic regression analyses with the discovery cohort dataset showed that the combination of CCL1, CXCL10, VEGF, and ADA2 (without CRP) yielded the lowest AIC (29.78) with area under the curve (AUC = 0.9525). The following formula was produced: 1(1+e^(−(−12.0204+(6.0145∗Log10CCL1)+(−3.0394∗log10CXCL10)+(3.4635∗Log10VEGF)+(0.9583∗ADA2)))) with optimal cutoff value set on 0.50998. Adding the values obtained for each marker per participant within the formula obtained a new value. If this value was greater than the optimal cutoff, the participant was classified as ATB. This model with optimal cutoff value yielded a sensitivity of 95% and specificity of 90% in distinguishing ATB patients from untreated LTBI individuals in the discovery cohort (**Table 3**, **Supplementary Figure S2**). For total ADA activity, the same marker combination gave the lowest AIC (AUC = 0.9475; **Table 3**). With the optimal cutoff set on 0.44997, this biomarker combination yielded a sensitivity of 100% and specificity of 90% in the discovery cohort. Patients with extrapulmonary disease were correctly identified as ATB by both biomarker signatures (data not shown). Together, these data indicate that a combination of markers may be a promising tool to distinguish ATB from untreated LTBI.

**Table 3:** Evaluation biomarker signature (CCL1, CXCL10, VEGF, and ADA2 or total ADA) in distinguishing ATB from untreated LTBI.

	CCL1, CXCL10, VEGF, and ADA2	CCL1, CXCL10, VEGF, and total ADA
AUC	0.9525	0.9475
**Coefficients**		
Intercept	-12.0204	-10.1068
Log10 CCL1	6.0145	6.4648
Log10 CXCL10	-3.0394	-3.5797
Log10 VEGF	3.4635	3.2062
ADA2	0.9583	NA
Total ADA	NA	0.5903
Optimal Cutoff	>0.5099783	>0.4499695
**Discovery cohort**		
Sensitivity (%)	95	100
Specificity (%)	90	90
**Leiden cohort**		
Sensitivity (%)	75	67
Specificity (%)	100	100
**Italy cohort**		
Sensitivity (%)	100	100
Specificity (%)	30	20

CCL, C-C motif chemokine ligand 1; CXCL10, C-X-C motif chemokine ligand 10; VEGF, vascular endothelial growth factor; ADA2, adenosine deaminase 2 activity; total ADA, total adenosine deaminase activity; AUC, area under the curve; NA, not applicable.

### Validation of Serum Biomarker Profiles for the Identification of ATB and Untreated LTBI

To confirm the usefulness of these biomarker signatures in discriminating ATB patients from untreated LTBI individuals, the signatures and corresponding optimal cutoff values were first applied on the dataset obtained with the samples originating from the Leiden cohort. In this cohort, the included participants in the untreated LTBI group were similar to the untreated LTBI group of the discovery cohort. Using the biomarker signature including ADA2 activity, the accuracy in distinguishing ATB from untreated LTBI was 90.6%, with sensitivity of 75% and specificity of 100%. The biomarker profile with total ADA activity yielded an accuracy of 87.5%, with sensitivity of 67% and specificity of 100%. Results are shown in **Table 3** and **Supplementary Table S3**.

In the Italian cohort, where untreated HH-LTBI individuals were sharing their households with ATB patients, we obtained an accuracy of 72% for the biomarker signature with ADA2 activity, a sensitivity of 100%, and specificity of 30%. The profile including total ADA activity yielded the same sensitivity; however, a specificity of 20% (**Table 3**, **Supplementary Table S3**).

Results obtained for both the discovery cohort and validation cohort (Leiden) indicated that the combinations of CCL1, CXCL10, VEGF, and ADA(2) activity could be promising tools for discriminating between ATB patients and untreated LTBI individuals. However, the model lacks sufficient specificity to discriminate ATB patients and untreated HH-LTBI individuals sharing a household with an ATB patient before inclusion in the study.

## Discussion

With currently available routine sputum-based tests, it may be difficult to differentiate ATB from LTBI. In daily practice, this means that ATB patients might be undertreated, possibly leading to severe morbidity and continued transmission due to untreated disease, whereas LTBI individuals might be overtreated with an unnecessary drug exposure and toxicity (2, 8). Therefore, the aim of this study was to identify novel biomarker profiles to differentiate ATB from LTBI using serum. Such a tool may help clinicians in deciding whether patients should receive full medical treatment in ATB patients without microbiological confirmation or prophylactic treatment in case of LTBI.

To our knowledge, this is the first study evaluating the diagnostic accuracy of activities of serum ADA and its isozymes in combination with other serum biomarkers to differentiate ATB from LTBI. In ATB patients, CCL1, VEGF, CRP, CXCL10, and ADA(2) activity were significantly elevated compared to untreated LTBI individuals. Increased concentrations of CCL1, CRP, VEGF, and CXCL10 in ATB compared to LTBI are in line with previous reports (30, 31, 38–41, 54) and may reflect the activity of infected macrophages, innate immune cells such as natural killer (NK) cells and innate lymphoid cells (ILCs), as well as activated T lymphocytes. CCL1, also known as I-309, is a glycoprotein secreted by activated T lymphocytes and stimulates chemotaxis of monocytes (55). VEGF and CXCL10 are produced by activated macrophages and antigen-presenting cells and regulate cell growth and chemotaxis and may be the driving forces for stimulated angiogenesis as observed in ATB lesions (56–58). CXCL10 may also limit *Mtb* replication, as recently observed (59, 60). CRP is synthesized in the liver and is secreted following interleukin (IL)-6 stimulation by macrophages and T lymphocytes (61). CRP levels have been shown to depend on the virulence of the *Mtb* strain and the location of the infection, for example, pulmonary TB and miliary TB show higher levels of CRP (62). Activation of monocytes during TB infection is further emphasized by increased ADA activity and in particular of ADA2. ADA is an enzyme that protects cells from apoptosis by eliminating intracellular toxic derivatives of both adenosine and deoxyadenosine (63). The isoform ADA2 is exclusively produced by monocytes and macrophages when exposed to, or infected by, intracellular bacterial pathogens, such as *Mtb (* 64). Secretion of ADA2 promotes CD4^+^ T-cell proliferation and differentiation of monocytes into macrophages followed by proliferation (63). The diagnostic utility of total ADA(2) activity has been studied in various TB (sub)populations, and increased total ADA activity was shown in serum from ATB patients when compared with HCs or cured TB subjects combined with positive TST (20, 65, 66). However, in these studies, no comparison was made between ATB patients and LTBI individuals.

Validating the combination of ADA(2) activity with CCL1, CXCL10, and VEGF in two independent cohorts led to confirmatory results, with some differences observed as well. The obtained sensitivity in identifying ATB was high in both validation cohorts. However, the specificity obtained within the Leiden cohort was high, but in the Italian cohort, it was low. As discussed before, within this Italian cohort, untreated HH-LTBI participants were individuals sharing their households with patients diagnosed with ATB not more than 3 months before inclusion in the study. We expect that close proximity with an ATB patient increases the exposure to *Mtb.* Therefore, we hypothesize that the Italian untreated LTBI individuals may have an active *Mtb* replication being in an intermediate stage between ATB and untreated LTBI. This difference may have led to incorrect identification of the Italian untreated LTBI patients using the biomarker profiles identified.

Wawrocki et al. (67) described serum IL18, IL37, and CXCL10 as potential biomarker signatures for distinguishing ATB from LTBI. In the current study, we did not measure IL37; however, IL18 was present in the top 10 markers that contribute to principal component 2 (**Figure 1**), suggesting that this marker may play a role in distinguishing ATB from untreated LTBI. However, in our cohort, other markers were found to be more promising in discriminating ATB from LTBI, including CXCL10.

A limitation of our designed prediction model is the lack of samples from patients with different diagnoses, such as pneumonia, lung cancer, sarcoidosis, vasculitis, non-tuberculous mycobacterial infections, and aspergillosis. Others reported ADA(2) activity in populations of ATB, pneumonia, lung cancer, and pleural effusions of any origin with sensitivity and specificity values of ADA(2) ranging from 35% to 94% and 55% to 97%, respectively (19, 20, 68–70). In addition, both CXCL10 and VEGF have been described to be potential biomarkers for various diagnoses such as chronic obstructive pulmonary disease (COPD) and non-small cell lung cancer and for exacerbations of autoimmune diseases. The absence of a subanalysis for endemic background might be another limitation of this study. However, when comparing individuals from a high-burden country to participants from a low-burden country in the ATB and untreated LTBI groups of the discovery cohort, ADA and cytokine/chemokine measurements are not significantly different (p ≥ 0.18, data not shown).

The lack of a gold standard for LTBI is a known limitation. Until diagnostic tools are available to detect the presence of live *Mtb* in LTBI, we are dependent on the WHO definition for LTBI (2). To get better insight in LTBI individuals, we included two LTBI subgroups in the discovery cohort, which provides added value compared to several other studies (33, 42, 56, 71, 72). Treated LTBI individuals are assumed to be “cured,” and any difference between both LTBI groups possibly could indicate which of the untreated LTBI individuals represent persistent infection. Unfortunately, results of untreated LTBI and treated LTBI were highly overlapping in our cohorts and did not show any significant differences.

In conclusion, the combination of CCL1, CXCL10, VEGF, and ADA(2) activity in serum resulted in promising signatures able to differentiate between ATB and LTBI. These obtained biomarker signatures could facilitate the ongoing challenge of diagnosing TB and distinguishing ATB from LTBI. More research is necessary to optimize the prediction models in prospective clinical studies and including geographically different populations.

## Data Availability Statement

The original contributions presented in the study are included in the article/**Supplementary Material**, further inquiries can be directed to the corresponding author.

## Ethics Statement

The studies involving human participants were reviewed and approved by Medical Research Ethics Committees-United (NL53628.100.15) and the Board of Directors of the Public Health and Diakonessenhuis, Utrecht, Netherlands. The patients/participants provided their written informed consent to participate in this study.

## Author Contributions

ED and LH processed the experimental data, drafted the article, and designed the figures. AB, LK, J-WL, ST, RH, and SN designed the study. ED, LH, and JD performed data analyses. SJ, TO, OA, DG, EP, AN, IH, and RH provided resources. BB, SP, and RH contributed to sample preparation and data analyses. All authors contributed to the article and approved the submitted version.

## Funding

This work was supported by the Italian Ministry of Health, Ricerca Corrente, Linea 4 and Italian Ministry of Health, GR-2018-12367178.

## Conflict of Interest

The authors declare that the research was conducted in the absence of any commercial or financial relationships that could be construed as a potential conflict of interest.

The reviewer HD declared a past co-authorship with the authors SJ and TO to the handling editor.

## Publisher’s Note

All claims expressed in this article are solely those of the authors and do not necessarily represent those of their affiliated organizations, or those of the publisher, the editors and the reviewers. Any product that may be evaluated in this article, or claim that may be made by its manufacturer, is not guaranteed or endorsed by the publisher.
