# Supplementary material for: Serum Biomarker Profile Including CCL1, CXCL10, VEGF, and Adenosine Deaminase Activity Distinguishes Active From Remotely Acquired Latent Tuberculosis
Source: Front Immunol. 2021 Oct 7;12:725447. doi: 10.3389/fimmu.2021.725447 (PMC8529994; doi:10.3389/fimmu.2021.725447)
Supplement: Supplementary file 2 [file DataSheet_2.docx]

**Supplementary Material**

**Supplementary Table 1.** **Cytokine concentrations (pg/mL) after imputation and ADA activity levels (U/L) in individuals diagnosed with ATB, untreated-LTBI, treated-LTBI and healthy controls from discovery cohort.**

|  | ATB  Median (IQR)  (n=20) | Untreated-LTBI Median (IQR)  (n=20) | Treated-LTBI  Median (IQR)  (n=20) | HC  Median (IQR)  (n=20) |
| --- | --- | --- | --- | --- |
| ACE | 2.37x10^5^  (1.36x10^5^-2.89x10^5^) | 2.29x10^5^  (1.86x10^5^-2.45x10^5^) | 2.06x10^5^  (1.79x10^5^-2.67x10^5^) | 2.14x10^5^  (1.75x10^5^-2.35x10^5^) |
| CD40L | 3894.06  (2810.69- 4428.86) | 3185.81  (2101.61-4256.16) | 3393.86  (2106.73-4449.17) | 3073.05  (2262.70-3730.27) |
| CRP | 2.11x10^7^  (1.53x10^7^-3.33x10^7^) | 2.25x10^6^  (6.85x10^5^-8.84x10^6^) | 7.74x10^5^  (4.34x10^5^-3.74x10^6^) | 6.88x10^5^  (4.07x10^5^-2.12x10^6^) |
| EGF | 76.37  (29.62-113.57) | 64.79  (23.95-120.66) | 96.98  (35.20-128.08) | 91.44  (71.45-118.68) |
| Elastase | 2.81x10^4^  (2.47x10^4^-3.31x10^4^) | 2.72x10^4^  (2.36x10^4^-3.15x10^4^) | 2.71x10^4^  (1.86x10^4^-3.13x10^4^) | 2.47x10^4^  (1.95x10^4^-3.14x10^4^) |
| FASL | 8.70  (8.70-8.70) | 8.70  (8.70-8.70) | 8.70  (8.70-8.70) | 8.70  (8.70-8.70) |
| Galectin-9 | 2911.23  (2317.53-4030.44) | 2353.20  (1682.09-3733.72) | 1983.95  (1600.60-2310.39) | 1977.98  (1379.65-2854.12) |
| Granzyme B | 20.25  (20.25-20.25) | 20.25  (20.25-41.95) | 20.25  (20.25-20.25) | 20.25  (20.25-73.64) |
| HSPA1 | 97.65  (97.65-97.65) | 97.65  (97.65-97.65) | 97.65  (97.65-97.65) | 97.65  (97.65-97.65) |
| IFNG | 4.40  (4.40-4.40) | 4.40  (4.40-15.21) | 4.40  (4.40-4.40) | 4.40  (4.40-36.00) |
| IL1Ra | 17.11  (4.20-135.59) | 49.24  (35.10-152.89) | 4.20  (4.20-127.06) | 52.88  (11.93-123.17) |
| IL1A | 8.42  (3.52-11.61) | 8.07  (5.91-12.49) | 7.42  (3.95-10.80) | 7.10  (4.27-7.69) |
| IL1B | 0.60  (0.60-0.60) | 0.60  (0.60-0.60) | 0.60  (0.60-0.60) | 0.60  (0.60-0.60) |
| IL2 | 2.10  (2.10-2.10) | 2.10  (2.10-2.10) | 2.10  (2.10-2.10) | 2.10  (2.10-2.10) |
| IL6 | 1.29  (0.30-1.40) | 1.40  (0.72-1.40) | 1.40  (0.51-1.40) | 1.26  (0.34-1.40) |
| CXCL8 | 17.31  (13.94-30.13) | 16.19  (4.67-29.27) | 17.06  (13.01-28.28) | 13.54  (8.47-32.32) |
| IL10 | 5.97  (1.98-10.97) | 8.43  (1.70-21.71) | 2.10  (1.70-10.15) | 5.67  (1.78-35.43) |
| IL12 | 3.30  (3.30-3.30) | 3.30  (3.30-8.27) | 3.30  (3.30-3.30) | 3.30  (3.30-3.30) |
| IL13 | 3.00  (2.98-3.00) | 3.00  (3.00-20.59) | 3.00  (3.00-3.00) | 3.00  (3.00-3.00) |
| IL15 | 5.95  (1.50-11.15) | 6.45  (1.50-13.02) | 1.50  (1.50-8.01) | 1.92  (1.50-9.00) |
| IL17 | 2.85  (2.85-2.85) | 2.85  (2.85-2.85) | 2.85  (2.85-2.85) | 2.85  (2.85-2.85) |
| IL18 | 127.39  (97.25-191.00) | 99.92  (82.25-205.96) | 85.08  (56.83-118.36) | 58.75  (45.66-86.26) |
| IL21 | 8.05  (8.05-82.41) | 8.05  (8.05-1207.41) | 8.05  (8.05-130.80) | 8.05  (8.05-854.62) |
| IL22 | 1.85  (1.85-1.85) | 1.85  (1.85-1.85) | 1.85  (1.85-1.85) | 1.85  (1.85-1.85) |
| IL25 | 82.15  (82.15-82.15) | 82.15  (82.15-82.15) | 82.15  (82.15-82.15) | 82.15  (82.15-82.15) |
| IL33 | 5.50  (5.50-5.50) | 5.50  (5.50-79.82) | 5.50  (5.50-5.50) | 5.50  (5.50-5.50) |
| CXCL10 | 557.78  (294.88-927.94) | 203.74  (154.23-415.95) | 196.18  (149.54-276.06) | 190.88  (149.72-238.84) |
| CCL1 | 21.71  (15.35-24.84) | 4.99  (2.15-8.48) | 4.31  (2.15-7.18) | 3.68  (2.15-6.54) |
| CCL2 | 94.26  (68.31-137.20) | 108.71  (81.96-137.92) | 108.20  (84.64-154.49) | 112.12  (76.98-130.30) |
| CCL8 | 27.38  (17.86-58.01) | 46.83  (32.40-62.21) | 38.74  (28.51-59.67) | 39.95  (35.56-51.27) |
| CCL22 | 516.46  (411.85-1031.71) | 621.21  (394.76-869.55) | 765.98  (625.65-919.68) | 657.93  (504.90-921.87) |
| CXCL9 | 9.03  (4.53-12.45) | 2.34  (2.10-8.81) | 2.10  (2.10-3.02) | 2.10  (2.10-2.88) |
| CCL4 | 67.65  (51.04-83.75) | 64.53  (45.82-105.30) | 80.81  (50.96-103.81) | 62.97  (52.36-85.79) |
| SAA1 | 593.75  (593.75-593.75) | 593.75  (593.75-593.75) | 593.75  (593.75-593.75) | 593.75  (593.75-593.75) |
| sIL2RA | 282.61  (168.77-448.57) | 203.99  (29.15-298.85) | 171.46  (35.66-287.28) | 133.52  (65.39-245.09) |
| S100A8 | 2110.64  (1322.19-3108.84) | 1447.10  (944.98-2776.85) | 1803.82  (1196.87-3653.71) | 2171.70  (1074.25-2881.19) |
| CCL17 | 262.33  (165.95-559.02) | 219.61  (103.16-302.23) | 202.58  (103.22-268.36) | 183.20  (109.11-260.77) |
| TNF | 1.10  (1.10-1.10) | 1.10  (1.10-1.10) | 1.10  (1.10-1.10) | 1.10  (1.10-1.10) |
| TNFSF10 | 24.98  (11.14-50.02) | 44.26  (7.20-57.36) | 36.54  (7.20-50.32) | 29.50  (7.48-39.88) |
| VEGF | 315.57  (206.86-441.40) | 152.07  (86.57-278.41) | 186.08  (95.27-298.70) | 199.34  (155.27-281.98) |
| Total ADA activity* | 9.11  (7.29-12.26) | 5.45  (3.95-8.25) | 5.60  (4.13-7.68) | 4.79  (4.00-7.18) |
| ADA1 activity* | 2.82  (1.94-4.25) | 2.11  (1.58-3.23) | 2.40  (1.59-3.00) | 2.25  (1.62-2.61) |
| ADA2 activity* | 6.62  (4.89-8.70) | 3.07  (2.13-5.10) | 3.15  (2.50-4.45) | 2.94  (2.13-4.68) |

ATB = active tuberculosis, IQR = interquartile range, untreated-LTBI = latent tuberculosis infection without prophylaxis, treated-LTBI = latent tuberculosis infection after completion of prophylaxis, HC = healthy control, CRP= C-reactive protein, EGF = epidermal growth factor, FASLG = FAS-ligand, HSPA1 = heat shock family A (HSP70) member 1, IFNG = interferon gamma, IL1RN = IL1 receptor antagonist, CXCL8 = C-X-C motif chemokine ligand 8, CXCL10 = C-X-C motif chemokine ligand 10, CCL1 = C-C motif chemokine ligand 1, CCL2 = C-C motif chemokine ligand 2, CCL8 = C-C motif chemokine ligand 8, CCL22 = C-C motif chemokine ligand 22, CXCL9 = C-X-C motif chemokine ligand 9, CCL4 = C-C motif chemokine ligand 4, SAA1 = serum amyloid A1, IL2RA = IL2 receptor subunit alpha, S100A8 = S100 calcium binding protein A8, CCL17 = C-C motif chemokine ligand 17, TNF = tumor necrosis factor alpha, TNFSF10 = tumor necrosis factor superfamily member 10, VEGF = vascular endothelial growth factor, ADA = adenosine deaminase, ADA1 = adenosine deaminase 1, ADA2 = adenosine deaminase 2, * This marker was measured in 9 healthy controls.

**Supplementary Table 2. Evaluation of diagnostic accuracy of the explored markers: total ADA activity, ADA2 activity, CCL1, CRP, CXCL10 and VEGF separate in differentiating ATB from untreated-LTBI in discovery cohort.**

|  | **ADA2 activity** | **Total ADA activity** | **CCL1** | **CRP** | **CXCL10** | **VEGF** |
| --- | --- | --- | --- | --- | --- | --- |
| **AUC** | 0.86 | 0.81 | 0.87 | 0.86 | 0.80 | 0.75 |
| **AIC** | 41.4 | 45.2 | 40.0 | 38.9 | 49.3 | 50.3 |
| **Cut-off** | > 0.399 | > 0.409 | > 0.619 | > 0.682 | > 0.337 | > 0.356 |
| **Sensitivity (%)** | 90 | 90 | 85 | 80 | 95 | 90 |
| **Specificity (%)** | 75 | 65 | 85 | 85 | 60 | 55 |

ADA = adenosine deaminase, ADA2 = adenosine deaminase 2, CCL = C-C motif chemokine ligand 1, VEGF = vascular endothelial growth factor, CRP = C-reactive protein, CXCL10 = C-X-C motif chemokine ligand 10, ATB = active tuberculosis, untreated-LTBI = latent tuberculosis infection without prophylaxis, AUC = area under the curve.

* measured in U/L, ** measured in pg/ml.

**Supplementary Table 3. Prediction accuracy of diagnosis of ATB and untreated-LTBI individuals/households in both validation cohorts.**

| **Biomarker signature including ADA2** | | | | | | | | | | |
| --- | --- | --- | --- | --- | --- | --- | --- | --- | --- | --- |
|  | | Original diagnosis | | |  |  | | Original diagnosis | | |
|  | Leiden cohort | ATB | Untreated-LTBI | Total |  |  | Italy cohort | ATB | Households | Total |
| Predicted diagnosis | ATB | 9 | 0 | 9 |  | Predicted diagnosis | ATB | 30 | 14 | 44 |
|  | Untreated-LTBI | 3 | 20 | 23 |  |  | Households | 0 | 6 | 6 |
|  | Total | 12 | 20 |  |  |  | Total | 30 | 20 |  |
| **Biomarker signature including total ADA** | | | | | | | | | | |
|  | | Original diagnosis | | |  |  | | Original diagnosis | | |
|  | Leiden cohort | ATB | Untreated-LTBI | Total |  |  | Italy cohort | ATB | Households | Total |
| Predicted diagnosis | ATB | 8 | 0 | 8 |  | Predicted diagnosis | ATB | 30 | 16 | 46 |
|  | Untreated-LTBI | 4 | 20 | 24 |  |  | Households | 0 | 4 | 4 |
|  | Total | 12 | 20 |  |  |  | Total | 30 | 20 |  |

The biomarker signature formula’s (CCL1, CXCL10, VEGF and ADA2 activity or total ADA activity) and optimal cut-off’s obtained via the discovery cohort, were used to predict the diagnosis of patients included in both validation cohorts.

**Supplementary figure legends**

**Supplementary Figure 1. Principal Component analysis of Pulmonary and Extrapulmonary ATB patients.**Plots of the first two principal components (A) and the second and third principal components (B) reveal no separation between Pulmonary (grey dots) and Extrapulmonary (black dots) ATB patients.

**Supplementary Figure 2. Receiver operating characteristic (ROC) curve depicting the sensitivity and specificity of biomarker profiles including CCL1, CXCL10, VEGF and ADA2 activity or total ADA activity in differentiating ATB from untreated-LTBI obtained with the discovery cohort.** The black line represents the biomarker signature including CCL1, CXCL10, VEGF and ADA2 activity, the dotted line represents the signature including CCL1, CXCL10, VEGF and total ADA activity, the diagonal line represents the reference line. AUC ADA2= Area under the curve obtained with biomarker signature including ADA2 activity. AUC Total ADA = Area under the curve obtained with biomarker signature including total ADA activity.
