# Supplementary figures and images for: Serum Biomarker Profile Including CCL1, CXCL10, VEGF, and Adenosine Deaminase Activity Distinguishes Active From Remotely Acquired Latent Tuberculosis
Source: Front Immunol. 2021 Oct 7;12:725447. doi: 10.3389/fimmu.2021.725447 (PMC8529994; doi:10.3389/fimmu.2021.725447)

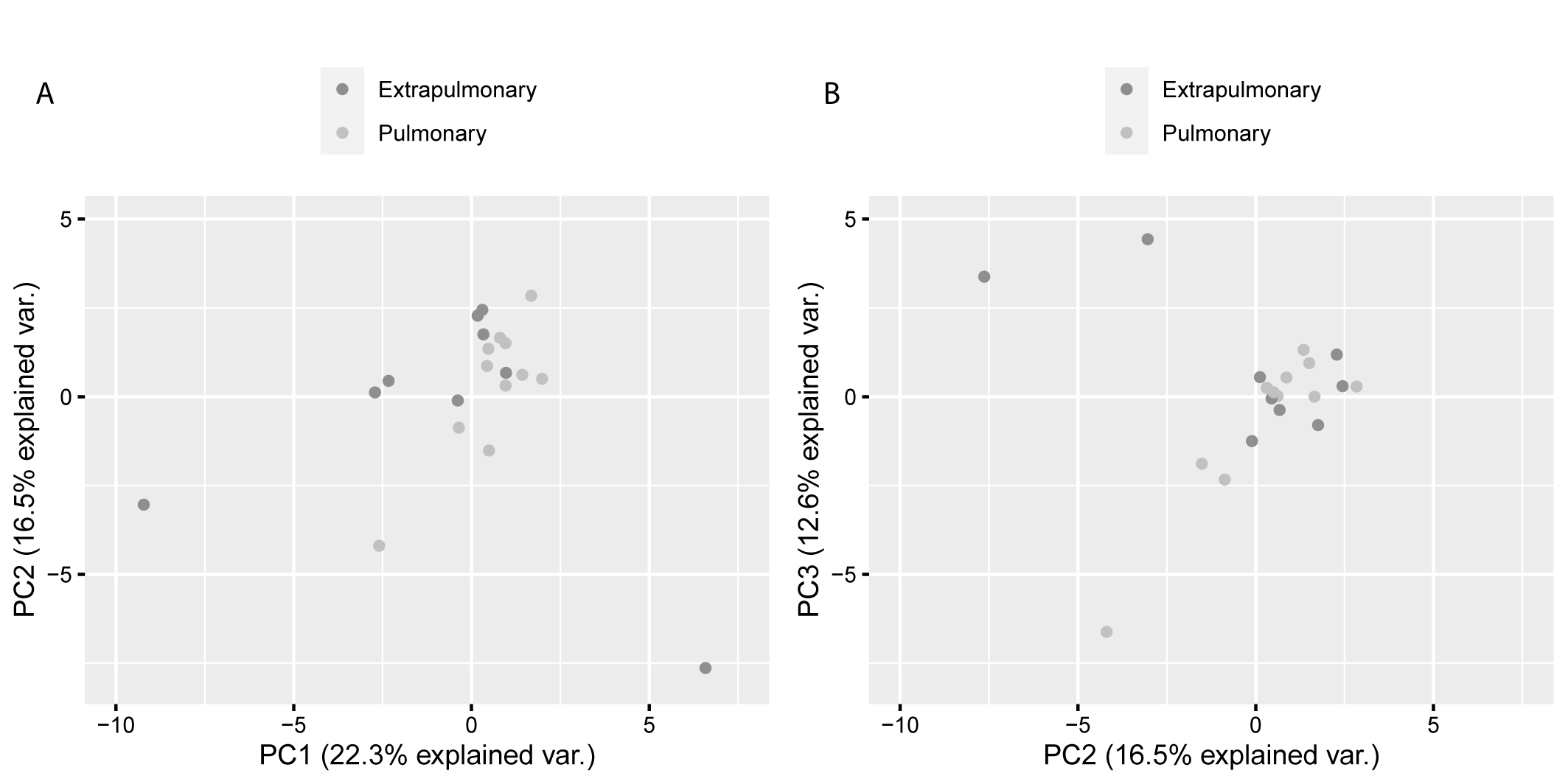

Supplement: Supplementary file 3 [file Image_1.tif]

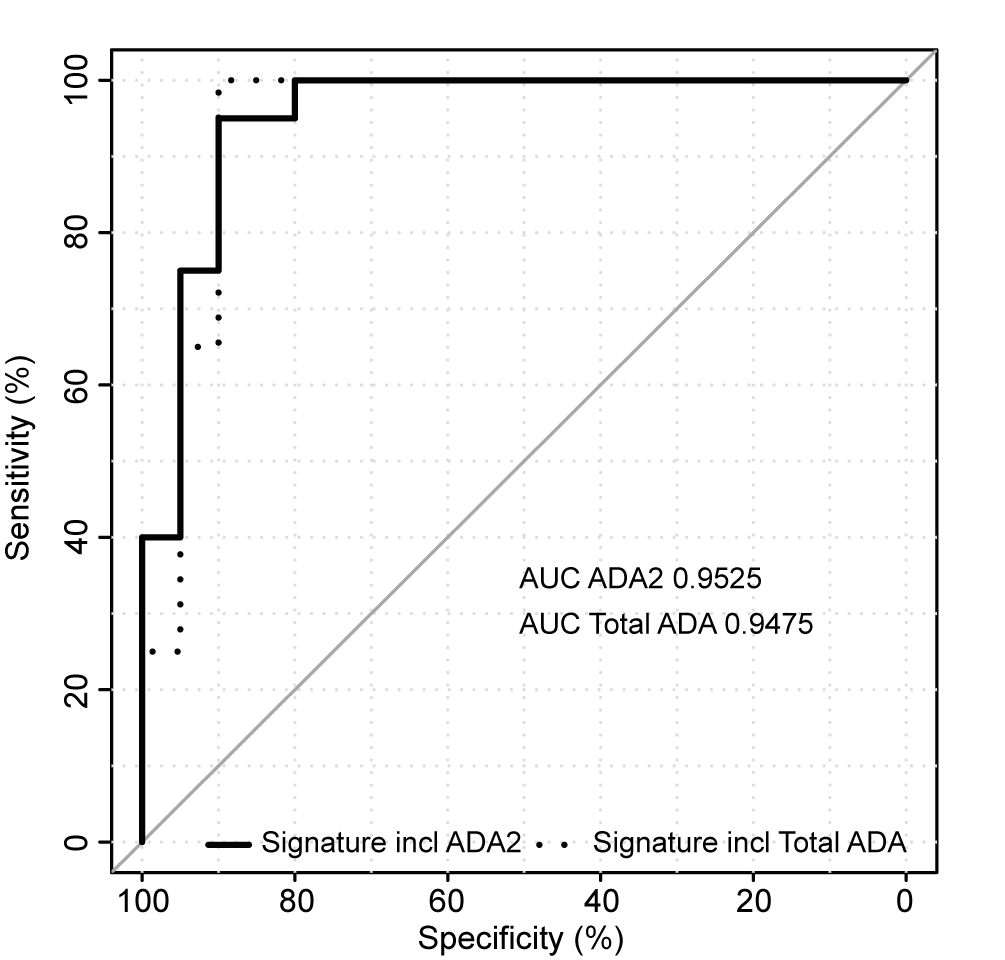

Supplement: Supplementary file 4 [file Image_2.tif]
